# Neurological abnormalities among pediatric patients with sickle cell disease in Saudi Arabia: a single-center retrospective study

**DOI:** 10.3389/fped.2023.1290314

**Published:** 2024-01-10

**Authors:** Ziad T. Basuni, Dania A. Monagel, Areej Taha, Nehal Ahmed, Amany Ahmed

**Affiliations:** ^1^ Department of Oncology, Ministry of the National Guard- Health Affairs, Jeddah, Saudi Arabia; ^2^ King Abdullah International Medical Research Center, Jeddah, Saudi Arabia; ^3^ College of Medicine, King Saud bin Abdul-Aziz University for Health Sciences, Jeddah, Saudi Arabia

**Keywords:** sickle cell disease, pediatric, neurological complications, stroke, seizure, transient ischemic attack, high hemoglobin F

## Abstract

**Introduction:**

Sickle cell disease (SCD) is a common inherited blood disorder characterized by the production of abnormal sickle-shaped red blood cells. SCD can lead to various complications including neurological issues. Early detection and treatment are crucial for preventing these complications. This study aimed to describe the neurological manifestations, radiological findings, and neurological diagnosis related to SCD in Saudi children with the aim of contributing to the formulation of population-based guidelines for screening and treating SCD-related neurological complications.

**Methods:**

This descriptive retrospective study included pediatric patients aged < 14 years diagnosed with SCD who were regularly followed up at the hematology clinic in KAMC, Jeddah, Saudi Arabia, from January 2008 to January 2022. Demographic and clinical data were collected from the clinical charts of 101 participants.

**Results:**

This study included 101 patients with SCD with a mean age of 23 months at diagnosis. Among these, 59% had SCD and high fetal hemoglobin (HbF) levels. Neurological sequelae, including seizures, stroke, and other abnormalities, were observed in 26.7% of patients. There were no significant differences in the onset of neurological issues between the patients with SCD-high HbF and those with other SCD phenotypes.

**Discussion:**

This study highlights the increased risk of brain injury and neurocognitive deficits in children with SCD. The occurrence of neurological sequelae in many patients emphasizes the need for early detection and intervention. Some patients experience neurological complications despite having high HbF levels, suggesting that further interventions are needed. This study has some limitations, including its small sample size and retrospective nature.

**Conclusion:**

Early detection and intervention are crucial for neurological complications in patients with SCD. This study emphasizes the need for further research and effective treatment strategies considering the presence of neurological complications despite the presence of high HbF levels. Large-scale studies and population-specific guidelines are warranted for better understanding and management of SCD-related neurological complications in the Saudi population.

## Background

Sickle cell disease (SCD) is one of the most common single-gene mutations worldwide (1). Mutation in the beta-globin gene leads to the production of sickle hemoglobin, which has a reduced solubility compared to regular fetal (HbF) or adult hemoglobin (HbA). This contributes to hemoglobin polymerization and abnormal red blood cell shapes, resulting in vessel blockage and ischemia-reperfusion injury (2). Individuals with either a homozygous disease state or a compound heterozygous state involving sickle hemoglobin (HbS) are generally affected by SCD (2).

Molecular analysis of patients with sickle cell anemia SCD revealed the existence of significant haplotypes, including African and Arab-Indian (AI) haplotypes, also known as the Saudi haplotype. The AI haplotype is commonly found in the eastern region of Saudi Arabia and other Arab Gulf countries. Although the genetic abnormality responsible for the sickle cell gene is the same in all haplotypes, there is considerable variability in the clinical presentation and severity of the disease (3). Previous studies indicated that African haplotypes exhibit more severe and clinically significant manifestations of SCD, whereas individuals with the AI haplotype typically experience milder clinical symptoms (4, 5).

In Saudi Arabia, two prevalent haplotypes have been observed: the AI and Benin (African) haplotypes (6). Studies have indicated that individuals with the AI haplotype typically exhibit higher HbF levels and tend to experience milder symptoms of SCD, although the disease is not asymptomatic in these cases (7).

SCD is a complex disorder that affects multiple body systems and leads to high morbidity and mortality rates. Neurological complications are common and severe in SCD. This disease can cause neurological problems including stroke, seizures, transient ischemic attack (TIA), posterior reversible encephalopathy (PRES), moyamoya syndrome, silent cerebral infarcts, cognitive impairment, and neuropathic pain (8).

A retrospective cohort study of 189 newborns screened and diagnosed with SCD found that silent cerebral infarction was the most common cause of permanent neurological injury in children and adults with sickle hemoglobin (HbSS) and hemoglobin beta thalassemia (HbSb^0^ thalassemia). By the age of 18 years, approximately 39% of the children had experienced a silent cerebral infarct (9). Both stroke and silent cerebral infarcts are associated with significant cognitive impairment, resulting in an average 5-point decrement in full-scale IQ scores and negative impacts on educational attainment, employment status, and overall quality of life (8, 10). Children with SCD are at a higher risk of stroke, especially if they have abnormal transcranial doppler ultrasound (TCD) test results. Therefore, early detection and treatment are critical for preventing neurological complications of SCD (11).

To the best of our knowledge, there are no Saudi national data regarding the neurological outcomes in pediatric patients with SCD. In this retrospective study, we reviewed the neurological manifestations, radiological findings, and neurological complications related to SCD in children. We hypothesized that Saudi children with SCD would have higher HbF concentrations. We performed a subgroup analysis in patients with SCD associated with high HbF to determine if they have a lower rate of neurological sequelae due to the effect of circulating HbF. In future, our results could be combined with other available evidence to formulate population-based guidelines for the screening and treatment of SCD-related neurological complications.

## Methods

This descriptive retrospective study included all patients who were diagnosed with (HbSS, HbSS with high HbF, HbS-β thalassemia, and HbSC) and were younger than 14 years of age (as this is the upper age limit for the pediatric age group in our hospital). The group is regularly followed up at the hematology clinic at King Abdulaziz Medical City in Jeddah (KAMC-J) from January 2008 to January 2022.

A chart review was conducted between January 2021 and January 2022. Patients older than 14 years old, with sickle cell traits (HbAS), or lost to follow-up at KAMC-J were excluded. The research team reviewed the clinical charts of all the participants (*N* = 101) to collect demographic and clinical data using a Case Report Form (CRF) and the best care system. This study was approved by the King Abdullah International Medical Research Center (KAIMRC).

The Data collected included the patient's demographics, SCD diagnosis-related information (age at diagnosis since universal newborn screening for SCD is not available; SCD phenotype; genetics if available; and high HbF at initial diagnosis), and SCD management as hydroxyurea and transfusion history. Details of SCD complications, particularly neurological issues, were identified. Radiological images, including TCD and Magnetic resonance image (MRI), were reviewed. All patients with SCD in our clinic, regardless of genotype, underwent baseline TCD and MRI (can be performed without general anesthesia).

Our study included patients with SCD and high HbF levels as part of our subgroup analysis. Although there is no universally standardized definition of high HbF levels, Steinbergin *et al.* extensively reviewed the topic and found that HbF levels in SCD depend on several factors. However, they concluded that an HbF level of 20.7 ± 8.2% can be considered high (12). At our institution, a high HbF level was defined as 20% or higher.

## Statistical analysis

The analysis was performed using SPSS Statistics version 29 for Macintosh. Mean, median, range, and standard deviation (SD) were used for continuous variables, and frequency distributions were used to assess categorical data. The independent sample *t*-test, one-way ANOVA, and *χ*^2^ test were used to compare groups, and a *p*-value <0.05 was considered significant.

## Results

### Patient demographics

Between January 2021 and January 2022, data were collected from 101 patients who met the inclusion criteria. Of these, 58 were male and 43 were female. The mean age at diagnosis was 23 months (SD = 20) and that at data collection was 105 months (SD = 44). Based on Hb electrophoresis analysis before the initiation of hydroxyurea (HU) treatment 59 (58%) patients had HbSS with high HbF, 23 (22.8%) had HbSS disease, 18 (17.8%) had sickle beta thalassemia, and 1 (1%) had HbSC disease.

### Clinical characteristics

Five patients underwent whole exome sequencing (WES) in addition to SCD diagnosis, which revealed the following mutations: G6PD (*n* = 2), SPTA1 (*n* = 1), alpha thalassemia (*n* = 1), a compound heterozygous mutation causing sickle beta thalassemia (*n* = 1), and a FANCA variant of unknown significance (*n* = 1). WES is not a standard of care, but was performed in cases with other clinical indications.

**Figure 1:**
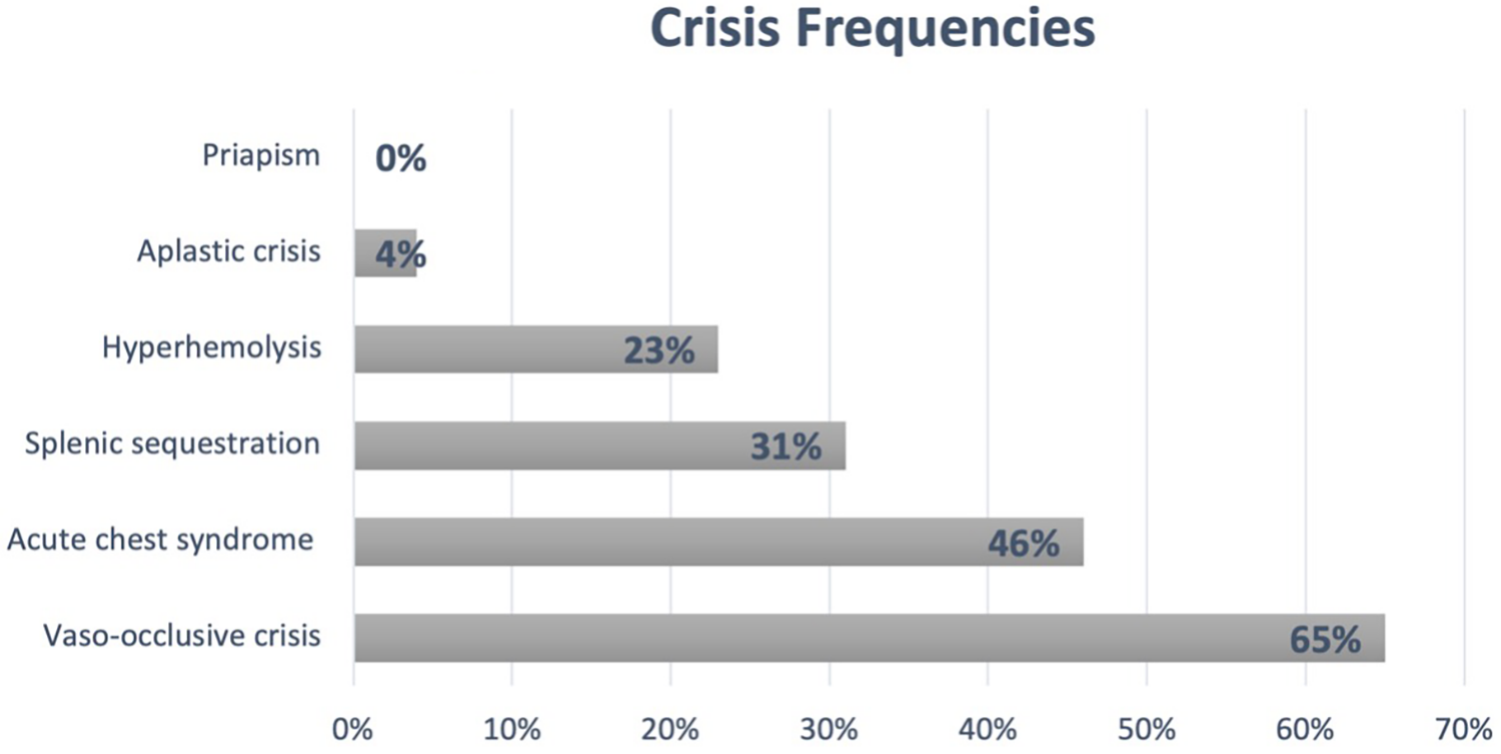
SCD crisis frequencies. Patients who experienced at least one episode.

Of the 101 patients included in the study, 81 (80%) underwent Hb electrophoresis at the time of diagnosis before starting HU; mean HbF: 26% (SD = 11.3) and HbS: 69% (SD = 10.3). Only 12 (11.8%) patients had no reported crises in the study center, with a mean number of admissions per patient of 6.4 (SD = 7.7) and an average of 0.8 admissions per patient per year (SD = 0.9). Additionally, 21% of patients had at least one admission to the pediatric intensive care unit. Figure 1 summarizes the most common SCD-related crises (other than neurological issues) in this cohort.

The mean Hb, white blood cell (WBC), neutrophil, and platelet counts prior to HU initiation were 8.9 g/dl (SD = 1), 12 × 10^9^/L (SD = 4.3), 4.3 × 10^9^/L (SD = 4.2), and 421 × 10^9^/L (SD = 192) respectively. Eighty-five patients underwent baseline TCD and 75 underwent at least one MRI/MRA.

The study found that 98% of participants (*n* = 99) were started on HU as a disease-modifying therapy, with a mean age at HU initiation of 46 months (SD = 32) and a mean maximum tolerated dose of HU of 21 mg/kg/day (SD = 9.3). Thirty percent of the patients required one or more exchange transfusions for different indications, including stroke, abnormal TCD, high hemoglobin level at the time of crisis, or preoperative preparation for any procedure, whereas 61% (*n* = 62) required simple blood transfusions, with 47% of the transfused group requiring five or more red blood cells (RBC) transfusions.

### Neurological sequelae

Of the 101 patients in the study, 27 (26.7%) had neurological sequelae (clinical or radiological), of whom 15 were male and 12 were female (Table 1). The mean age at sickle cell anemia diagnosis was 21 months (SD = 20) and 55% (*n* = 15) had sickle cell anemia with high HbF levels, 26% (*n* = 7) had HbSS disease, 15% (*n* = 4) had sickle beta thalassemia, and 3% (*n* = 1) had HbSC based on hemoglobin electrophoresis prior to starting HU.The mean Hb, WBC, neutrophil, and platelet counts before starting HU were 8.7 g/dl (SD = 1), 11.5 × 10^9^/L (SD = 3.3), 5.5 × 10^9^/L (SD = 6.8) and 471 × 10^9^/L (SD = 193), respectively. The mean age at the onset of neurological issues was 83 months (SD = 41), with 19% of affected children (*n* = 5) being ≤ 36 months old at the onset of the neurological abnormality. Eight patients had seizures, four had overt stroke, one had moyamoya syndrome, 13 had only radiological changes, one had sensory neuronal hearing loss, and one reported deterioration in school performance based on IQ assessment. The mean Hb, WBC, and platelet counts, and creatinine and urea levels at time of neurological insult were 8.7 g/dl (SD = 1.04), 11.7 × 10^9^/L (SD = 4.7), and 460 × 10^9^/L (SD = 207), and 36 µmol/L (SD = 8.2) and 4.4 mmol/L (SD = 9.3), respectively.

**Table 1:** Patients with neurological sequalae characteristics.

Patient	Gender	Age of diagnosis (months)	Baseline Hb (g/dl)	SCD phenotype	Clinical feature	Radiological finding	Treatment	Status at last follow up
1	F	13	10.5	SS + high Hb F	Seizure	TCD (N), MRI(N)	HU	Alive
2	M	12	9.7	SS + high Hb F	Deterioration of school performance (confirmed by neuropsych assessment)	TCD (N), MRI (N)	HU	Alive
3	M	7	9.5	SS + high Hb F	Seizure	TCD(N), MRI (N)	HU	Alive
4	F	12	7.9	SS + high Hb F	Seizure	TCD(N), MRI showed silent infarct	HU, Chronic transfusion	Alive
5	M	7	7	SS + high Hb F	Seizure	TCD (N), MRI (N)	HU	Alive
6	F	71	10.2	SS + high Hb F	Seizure	TCD (N), MRI(N)	HU	Alive
7	M	14	8.9	SS + high Hb F	Overt stroke, seizure, cognitive impairment	No TCD, MRI showed stroke	HU, Chronic transfusion	Dead
8	M	33	7.6	SS + high Hb F	Overt stroke	TCD (high), MRI showed stroke	HU, chronic transfusion, BMT	Alive
9	M	36	7.7	SS + high Hb F	None	TCD (N), MRI silent infarct	HU, Chronic transfusion	Alive
10	F	2	8.7	SS + high Hb F	None	TCD (N), MRI (silent infarct)	HU, Chronic transfusion, BMT	Alive
11	F	13	9.7	SS + high Hb F	None	TCD (low velocity)	HU	Alive
12	F	36	7.4	SS + high Hb F	SNHL	TCD (N) and MRI (N)	HU	Alive
13	F	28	8	SS + high Hb F	Seizure	TCD (High), MRI silent infarct	HU, Chronic transfusion	Alive
14	F	11	8.3	SS + high Hb F	None	TCD (N), MRI silent infarct/chairi malformation	HU, Chronic transfusion	Alive
15	M	33	9.8	SS + high Hb F	Speech delay	TCD (N), MRI irregularity A1 segment of ant cerebral artery	HU, BMT	Alive
16	M	9	10.3	SS	None	TCD (low velocities), MRI (N)	HU	Alive
17	F	0	8.7	SS	None	TCD (low velocities), MRI (N)	HU	Alive
18	M	48	8.8	SS	None	TCD (N), MRI showed silent infarct	HU, Chronic transfusion	Alive
19	M	84	9.2	SS	None	TCD (low velocity) MRI (N)	HU	Alive
20	F	7	8.4	SS	Overt stroke	High velocity on TCD, MRI showed stroke	HU, chronic transfusion	Alive
21	F	12	8.5	SS	Overt stroke, venous sinus thrombosis	High velocity on TCD, MRI (extensive venous sinus thrombosis and hemorrhagic stroke)	Chronic transfusion, BMT	Alive
22	M	4	8.7	SS	None	TCD (low velocity), MRI silent infarct	HU, chronic transfusion, BMT	Alive
23	M	0	8.8	S Beta thalassemia	None	TCD (low velocity), MRI silent infarct	HU, chronic transfusion	Alive
24	M	0	7.8	S Beta thalassemia	None	TCD (N), MRI showed moya moya	HU, chronic transfusion	Alive
25	M	3	8	S Beta thalassemia	None	TCD (N), MRI (silent infarct)	HU, chronic transfusion	Alive
26	M	19	8.5	S Beta thalassemia	None	TCD(N), MRI (silent infarct)	Chronic transfusion, HU	Alive
27	F	32	10.7	SC	Seizure	TCD (N), MRI (N)	HU	Alive

TCD, Transcranial doppler; MRI, magnetic resonance image; HU, Hydroxyurea; BMT, Bone marrow transplantation; (N), normal; SNHL, sensory neuronal hearing loss.

Chronic transfusion depends on the CBC, HbS level at time of the scheduled transfusion and can be either exchange transfusion or top up if the clinical and the lab parameter allowed.

All patients except one were receiving HU, with a mean age of 54 months (SD = 36 months) at initiation. The mean maximum tolerated dose of HU was 21 mg/kg/day (SD = 9). All patients with stroke, silent infarct, or abnormal TCD were placed in a chronic transfusion program, and four patients underwent bone marrow transplantation.

Although a significant proportion of patients with neurological injury had sickle cell-high HbF (56%), there was no statistically significant difference between patients with sickle cell-high HbF and those with other SCD phenotypes (*p* = 0.7). The mean age of onset of neurological diagnosis in children with sickle cell-high HbF ( months, SD = 42) and those with other SCD phenotypes ($\bar{x}=95$, SD = 37) was not significantly different [*t*(25) = −1.4, *p* = 0.9]. Table 2 summarizes the clinical and hematological profiles of patients with and without neurological complications.

**Table 2:** A comparison of the medical profile of children with SCD with and without neurological complication.

	Neurological Complication	No Neurological Complication	*p* value
Age at time of diagnosis (months), mean (SD)^b^	25.4 (19)	23 (20)	0.5
Gender^a^			0.8
M	16	42	
F	11	32	
High Hb F^a^			0.7
Yes	15	44	
No	12	30	
The use of hydroxyurea^a^			0.5
Yes	26	73	
No	1	1	
Age of starting HU (m), mean (SD)^b^	54 (36)	43 (30)	0.1
Max HU dose (mg/kg/day), mean (SD)^b^	20.9 (9)	20.5 (9.5)	0.7
Baseline hemoglobin (g/dl), mean (SD)^b^	8.7 (1)	8.9 (1)	0.8
Baseline Platelet ×10^9^/L, mean (SD)^b^	471 (193)	402 (189)	0.5
Baseline WBC ×10^9^/L, mean (SD)^b^	11.5 (3.3)	11.7 (4)	0.1
Number of admissions, mean (SD)^b^	10 (9)	5 (5.6)	0.019
Vaso-occlusive crisis^a^			0.5
Yes	19	47	
No	8	27	
PICU admission^a^			0.001
Yes	13	8	
No	14	66	

^a^ Chi squared test.

^b^ Independent sample *t*-test.

## Discussion

SCD predisposes children to an increased risk of developing brain injury (13). Patients with neurological insults develop a further decline in academic function and neurocognitive deficits, typically manifesting as executive dysfunction (14). The findings of this study provide a broad overview of the clinical and hematological profiles of Saudi children with SCD and highlight the occurrence of neurological sequelae in a significant proportion of patients.

SCD causes a broad range of neurological complications. Stroke is a significant contributor to morbidity and mortality in SCD, and The Cooperative Study of Sickle Cell Disease, which monitored approximately 4,082 adults and children over five years, showed a stroke prevalence of 3.75% with two peaks at 2–5 and >30 years. Interestingly, asymptomatic silent cerebral infarcts develop in 27% of patients before the age of 6 years and in 39% by 18 years of age. *Moyamoya syndrome* is a vasculopathy that develops in 30%–40% of patients and manifests at a young age. In addition, individuals with SCD have a higher risk of epilepsy. Nigeran *et al*. showed that seizures were 2–10 times more frequent in patients with SCD than in the general population. Other complications include PRES, paraplegia due to cord infarction, and venous sinus thrombosis (15, 16). In our cohorts, the most common manifestations of SCD were picked by routine surveillance (TCD followed by MRI) in asymptomatic participants, and in patients with seizures and stroke. Other documented complications include poor school performance, cerebral venous thrombosis, speech delay, and hearing loss. Most of our patients with neurological injuries received optimal supportive care measures, including HU, and there was only one death related to stroke and its complications. The study further highlights the role of HU, exchange transfusion, and simple blood transfusion in preventing further neurological damage in patients with SCD and neurological sequelae, as confirmed previously in international trials (11, 17). The study also identified various accompanying genetic mutations other than the sickle cell gene; although not routinely performed, it is important to consider genetic testing to identify other genes that may play a role in disease modification.

There is controversial evidence regarding the protective effects of HbF; by 5–10 years of age, children with SCD usually achieve stable HbF levels. The gene cluster in SCD has four main haplotypes: Bantu, Senegal, Benin, and AI. The highest HbF levels were found in the AI and Senegal haplotypes; these individuals usually have milder disease, although they can still be anemic and symptomatic. The modulatory effect of high HbF levels is induced by the inhibition of deoxy-HbS polymerization, which is one of the many functions of HbF. The HbF concentration in the blood does not represent HbF/F cells (F cells = number of cells with detectable HbF), meaning that HbF is not evenly distributed among F cells. Therefore, some cells have inadequate concentrations of HbF to protect against HbS polymerization, which explains why even patients with high HbF levels can still develop severe disease (18). In a cross-sectional study by Steinberg et al., high HbF in SCD was correlated with fewer pain crises and leg ulcers and lower occurrence rates of avascular necrosis and acute chest syndrome. Nonetheless, there was no apparent link to SCD nephropathy, blood pressure issues, priapism, stroke, or silent cerebral infarcts (19).

Furthermore, the majority of Saudi patients with SCD (AI haplotype) had high HbF levels and were likely to have milder disease. However, our data showed that even in this group, neurological complications (clinical or radiological) were observed in a significant number of patients. There were no statistically significant differences when comparing this finding between patients with high HbF levels and those with other SCD phenotypes. This finding suggests that the proposed protective effect of high HbF levels may not be sufficient to prevent neurological complications in patients with SCD and that the course of illness may not always be as benign as expected.

Clinical observations in Saudi patients with SCD by Sultan et al. demonstrated that the disease has a milder phenotype in children with HbF concentrations close to 30%, but symptoms become more prevalent in adults as the HbF concentration falls to 15%–20% (6). In contrast, we found that the mean age at SCD diagnosis was 21 months, which underscores the early onset of signs and symptoms of the disease, regardless of a high HbF status. Additionally, nearly one-fifth of the affected children were ≤ 36 months old at the onset of neurological abnormalities. This observation emphasizes that a high HbF level is one of our population's most potent genetic modifiers; however, other modifiers may counteract the effect of HbF and therefore further population-based studies are needed.

Neuropsychological assessments and sequelae in pediatric patients with SCD have not been sufficiently studied in our Saudi Patients with SCD. Gruntorad et al. had performed neurocognitive evaluations for 20 children with SCD and demonstrated significant neurocognitive deficits even in cases without major clinical or radiological changes. They highlighted that patients with SCD at increased risk not only for cognitive deterioration but also can suffer from other mental and psychological disorders such as attention-deficit hyperactivity disorder, adjustment disorder, depressed mood, impulse control, and conduct disorders (20).

Neurocognitive decline is usually linked to important illness-related factors, such as sickle cell genotype, neuroimaging findings, and the presence or absence of overt/silent infarcts. These children usually suffer from IQ deficits, attention/memory issues, and disorders of language/math/spelling/reading, with poor academic and career achievements overall. The availability of clinical neuropsychologists is extremely important in SCD programs. Their roles usually guide the selection of the most appropriate tools and answer pertinent questions while providing good insight into developmental considerations. Unfortunately, the lack such services in most of our centers affects multiple aspects of children's lives, including the proper transition into adulthood (21).

Overall, the findings of this study have important implications for the management and treatment of patients with SCD in Saudi Arabia and emphasize the need for continued research into effective treatment strategies to prevent or minimize the occurrence of neurological complications. In conclusion, this study provides valuable insights into the clinical and hematological profiles of patients with SCD and highlights the significant occurrence of neurological sequelae in affected patients. Despite the protective effects of high HbF levels, many patients still experience neurological complications, emphasizing the need for early detection and intervention. Future research should focus on a larger-scale multicenter national prospective study to better characterize the nature of neurological complications and develop a well-accepted national guideline for the Saudi population.

## Limitations

Our study had some limitations. First, it had a relatively small sample size (101 patients), which may limit the generalizability of the findings. Second, the study was conducted at a single center, which may limit the representativeness of the patient population. However, it is a tertiary care center that offers well-defined supportive care and bone marrow transplantation. Third, the lack of formal neurocognitive assessment is a significant limitation in assessing the population with SCD; in particular, it has a major impact on future performance. Unfortunately, our clinic as well is not based on multidisciplinary approach and the involvement of the neurologist is usually based on the case upon discretion of treating physician. Finally, this study relied on a retrospective review of medical records, which may have introduced bias or incomplete data.

## Data Availability

The datasets presented in this article are not readily available to maintain the confidentiality of our subjects. Requests to access the datasets should be directed to dania_monagel@hotmail.com.
